# Wear Properties of Conventional and High-Translucent Zirconia-Based Materials

**DOI:** 10.3390/ma15207324

**Published:** 2022-10-20

**Authors:** Francesco De Angelis, Matteo Buonvivere, Edoardo Sorrentino, Giuseppe Daniele Rondoni, Camillo D’Arcangelo

**Affiliations:** 1 Unit of Restorative Dentistry and Endodontics, Department of Medical, Oral and Biotechnological Science, School of Dentistry, “G. D’Annunzio” University of Chieti, 66100 Chieti, Italy; 2 Private Practice, 17100 Savona, Italy

**Keywords:** 3 mol% yttria-stabilized tetragonal zirconia polycrystal, 4 mol% yttria partially stabilized zirconia, 5 mol% yttria partially stabilized zirconia, gold alloy, wear

## Abstract

This study investigated the two-body wear resistance of a first generation 3 mol% yttria-stabilized tetragonal zirconia polycrystal (3Y-TZP), a second generation 3Y-TZP, a third generation 4 mol% yttria partially stabilized zirconia (4Y-PSZ), a 5 mol% yttria partially stabilized zirconia (5Y-PSZ), and a type III gold alloy (Aurocast 8), performed using opposing antagonistic cusps made out of the same material. Eight cylindrical specimens were prepared for each material (n = 8) for a total of forty specimens (N = 40). Conical cusps were fabricated for each material. Each cylinder–cusp pair was arranged inside a two-axis chewing simulator over up to 360,000 loading cycles. The wear resistance was analyzed by measuring the vertical substance loss (mm) and the volume loss (mm^3^). The antagonist wear (mm) was recorded before and after the wear test to evaluate the linear difference. Statistical analysis was performed using one-way analysis of variance (ANOVA); multiple comparisons were performed according to Tukey’s method. No statistically significant differences (*p* > 0.05) among the first generation 3Y-TZP, second generation 3Y-TZP, and 4Y-PSZ wear were found. 5Y-PSZ showed statistically significant higher wear compared to other the zirconias. Aurocast 8 displayed the highest values in terms of vertical wear, antagonist cusp wear, and volumetric loss. Although still not statistically comparable, the wear behavior of the latest 5Y-PSZ was the closest to the widely recognized gold standard represented by the type III gold alloy.

## 1. Introduction

An enormous selection of dental restorative materials have been launched on the market in an effort to improve biocompatibility [1], minimal invasiveness [2,3], and aesthetics in dentistry. Among the different mechanical features that characterize each material, wear behavior should be carefully evaluated by clinicians in an attempt to provide restorations mimicking the replaced tooth’s hard tissues [4]. Both a decreased wear resistance and an excessive hardness could, in fact, rapidly impair the aesthetic and functional outcomes achieved, especially in the full-mouth rehabilitation of parafunctional patients [5]. Gold-based dental alloys are well known in the literature for their enamel-like wear behavior, which, together with other desirable features, makes them especially suitable for durable posterior restorations [6,7]. However, their unpleasant appearance, paired with the increasingly pressing aesthetic demands of patients [8,9], has progressively reduced their clinical use in favor of all-ceramic materials such as zirconia [10,11].

Known for its exceptional mechanical features, 3 mol% yttria-stabilized tetragonal zirconia polycrystal (3Y-TZP), also called first generation zirconia, covers a wide range of clinical applications, from single crowns to multi-unit fixed dental prostheses and implants [12]. Owing to its inherent low translucency and opacity, however, its use is debatable in aesthetically critical areas [13], where silica-based feldspathic porcelains and glass-ceramics (within their mechanical limitations) still show superior aesthetical results in terms of natural enamel emulation [12]. Aiming at merging zirconia’s mechanical features and silica-based ceramics’ optical properties, over the past few years manufacturers have introduced several modifications to the first generation 3Y-TZP sintering procedure [14] and chemical/crystallographic structure [15], which have led to second generation 3Y-TZP, and third generation 4 mol% yttria partially stabilized zirconia (4Y-PSZ) and 5 mol% yttria partially stabilized zirconia (5Y-PSZ) [12]. Specifically, partially stabilized zirconias have initiated the next stage in monolithic zirconia development; characterized by a higher yttria content and an increased nonbirefringent cubic phase, these novel materials have shown noticeably improved translucency at the price of a decrease in flexural strength and toughness [15] and extended the original range of zirconia applications to include single crowns and multi-unit fixed dental prostheses in aesthetically demanding areas and even minimally invasive adhesive restorations (veneers, inlays/onlays) [12]. Several studies have investigated the bonding [16] and mechanical features of these materials [17], but only a few have addressed the issue of their wear behavior at present [18,19,20]. However, none of them have compared the wear resistance of these new materials with that of traditional zirconias and gold-based alloys. On these bases, the aim of the present article was to investigate and compare the wear behavior of a first generation 3Y-TZP, a second generation 3Y-TZP, a 4Y-PSZ, a 5Y-PSZ, and a type III gold alloy by means of a two-body wear test performed using opposing antagonistic cusps made out of the same restorative material. The null hypothesis was that no significant differences in terms of wear properties could be detected.

## 2. Materials and Methods

The experimental design of the study was planned considering the type of dental restorative material as the only qualitative factor under investigation, having five independent levels: first generation 3Y-TZP, second generation 3Y-TZP, 4Y-PSZ, 5Y-PSZ, and Aurocast 8.

A complete list of the materials used in the study is presented in Table 1.

### 2.1. Sample Fabrication Methodology

The sample size of the present experiment was based on similar previous studies [4,21,22,23]. Eight cylindrical specimens with an 8 mm diameter and a 6 mm height were prepared for each material (n = 8), for a total of forty specimens (N = 40). Zirconia cylinders (Katana Zirconia HTML, Katana Zirconia STML, Katana Zirconia UTML, and Zenotec Zr Bridge) were obtained by milling and sintering procedures according to the manufacturer’s instructions. Zenotec Zr Bridge specimens were sintered in a ceramic furnace (Programat S1 1600, Ivoclar Vivadent AG, Schaan, Liechtenstein) following the prescribed 10 h schedule (Program 21). All the other zirconia types were sintered in a conventional zirconia-sintering furnace (Noritake KATANA F-1; Kuraray Noritake, Tokyo, Japan) using the recommended 7 h schedule (1550 °C maximum temperature, 2 h hold time). Type III gold alloy specimens (Aurocast 8) were made through the classic lost wax technique. Wax replicas of the cylindrical specimens were produced, attached to wax sprue bases, and placed in a silicone casting ring. After the wax burnout process in a furnace (Magma No. 2300-0500; Renfert GmbH, Hilzingen, Germany), the hot ring was put in an automatic casting machine (ASM 30; Tecno-Gaz S.p.A, Sala Baganza, Italy).

### 2.2. Fabrication Methods of the Antagonist Model

Conical cusps with a 2 mm diameter round tip were fabricated for each of the materials analyzed. Zirconia cusps were obtained by the CAD/CAM technique from a pre-formed steel cusp scan, while gold alloy cusps were produced through the lost wax casting technique by taking a polyvinylsiloxane impression of the above-mentioned steel cusp to obtain its wax analogue.

### 2.3. Polishing Procedures

Zirconia cusps and cylindrical specimens were polished with silicon carbide rubbers (Pink Medium Midgets, RA #15; Dedeco International Inc, Long Eddy, NY, USA) and sandpaper cones (L-Red Meister Cones, Kuraray Noritake Dental Inc, Tokyo, Japan). Further polishing was performed using goat-hair brushes (RA Shiny S, Micerium S.p.A., Avegno, Italy) and a dedicated zirconia diamond paste (Pearl Surface Z, Kuraray Noritake Dental Inc.). Gold alloy cusps cylindrical specimens were polished using silicone polishers (Blue Fine and Pink Extra-Fine Midgets, HP #15, Dedeco International) and felt wheels with a specific diamond paste (Dia Past, NobilMetal S.p.A., Villafranca d’Asti, Italy). All polishing procedures were performed with an electric handpiece at about 15,000 rpm and manual pressure for 60 s.

### 2.4. Wear Testing

After a 24 h storage at 37 °C, each cylinder-cusp pair (manufactured using the same restorative material for both the cylinder and the cusp) was arranged inside a two-axis chewing simulator (CS-4.2, SD Mechatronik GmbH, Feldkirchen-Westerham, Germany) according to the methodology described by D’Arcangelo et al., acrylic resin (VariDur 200, Buehler, IL, USA) was used to fix the cylinders inside the chambers and the antagonist cusps in the corresponding holders. All cylinder–cusp pairs were then subjected to two-body wear test setting the parameters listed in Table 2. Specimens were subjected to a number of 360.000 cycles (corresponding to approximately 1.5 years of human mastication [24]) and kept submerged in artificial saliva for all the duration of the test.

### 2.5. Data Analysis

After wear testing, a three-dimensional analytical evaluation of the specimen wear facets was performed by quantitative surface assessment. Three-dimensional meshes of each cylindrical specimen were acquired through a CAD/CAM scanner (inEos X5, Dentsply Sirona, Charlotte, NC, USA) in STL (standard triangulation language) format, then converted to drawing interchange Format (DXF), and subsequently imported into a computer-aided design software (AutoCAD 2009, Autodesk Inc., San Rafael, CA, USA), taking care to choose a suitable triangular reference plane placed on the flat surface for depth measurement. Cusps were instead measured before and after the wear test to evaluate the detectable linear difference defined as antagonist wear (mm). Volume loss, as well as mean values, and standard deviation for wear depth were calculated using the SPSS Statistics 24 (IBM Corp., Armonk, NY, USA) statistical software. Mean values were compared using analysis of variance (ANOVA); multiple comparisons were performed according to Tukey’s method.

### 2.6. SEM Wear Facet Analysis

Following the measurements described above, the specimens were observed under a scanning electron microscope (SEM) (EVO 50 XVP LaB6, Carl Zeiss SMT Ltd., Cambridge, UK) following the metallization process, except for the gold alloy specimens. Surfaces were measured at 500× magnification so that it was possible to evaluate the wear surfaces of the cylindrical specimens examined. The SEM settings were as follows: high vacuum (2107 Torr), current output at 10 pA, accelerating voltage 10 kV, and a working distance of about 10 mm.

**Figure 1:**
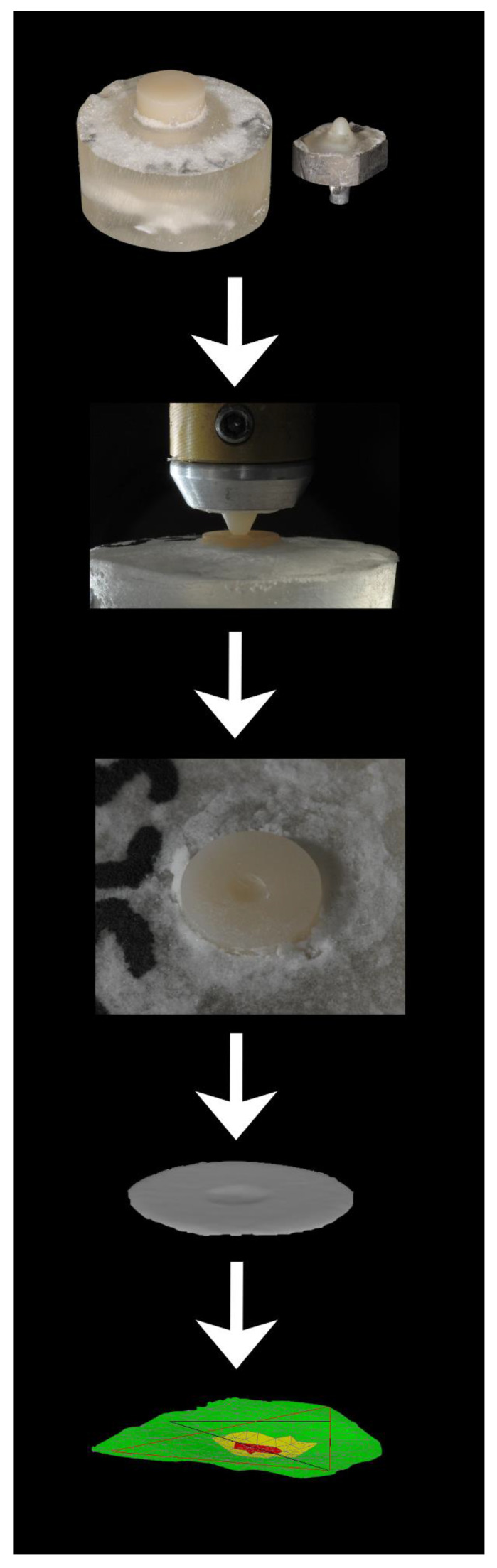
Experimental workflow from sample preparation to 3D analytical evaluation of the specimens’ wear facets.

The experimental workflow from sample preparation to 3D analytical evaluation of the specimens’ wear facets is summarized in Figure 1.

## 3. Results

The obtained results are summarized in Table 3.

One-way ANOVA tests revealed statistically significant differences (*p* < 0.05) between the groups regarding the parameters investigated. Multiple comparisons according to Tukey’s method showed no statistically significant differences (*p* > 0.05) among the first generation 3Y-TZP, second generation 3Y-TZP, and 4Y-PSZ wear values; significant differences (*p* < 0.05) were instead registered between 5Y-PSZ zirconia and all the other zirconia values. The 5Y-PSZ zirconia showed reduced antagonist cusp wear (0.115 ± 0.021 mm) and volumetric loss (0.067 ± 0.029 mm^3^), but similar vertical wear (0.073 ± 0.023 mm) compared to Aurocast 8, which displayed the highest value in terms of vertical wear (0.082 ± 0.017 mm), volumetric loss (0.131 ± 0.055 mm^3^), and antagonist cusp wear (0.238 ± 0.078 mm).

**Figure 2:**
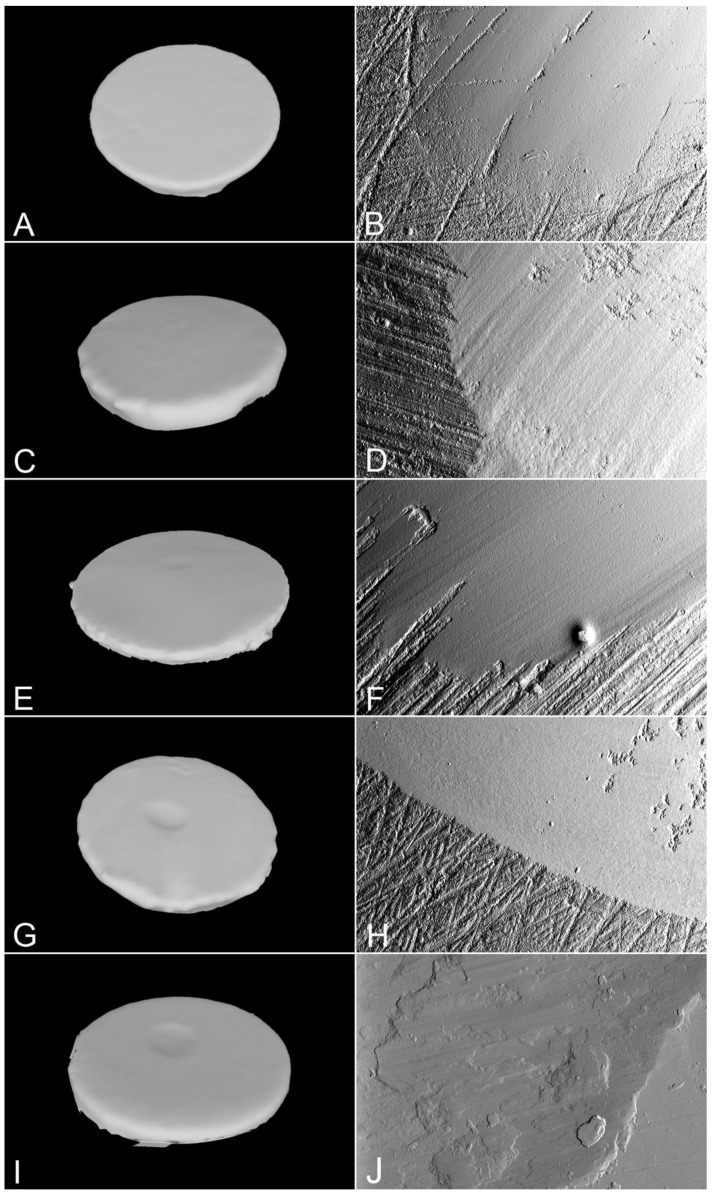
Three-dimensional meshes and scanning electron microphotographs (original magnification 500×) of representative first generation 3Y-TZP (**A**,**B**), second generation 3Y-TZP (**C**,**D**), 4Y-PSZ (**E**,**F**), 5Y-PSZ (**G**,**H**), and type III gold alloy specimens (**I**,**J**).

The 3D meshes and relative SEM analysis of the representative specimens are depicted in Figure 2. All the zirconia samples (Figure 2A–H) showed relatively flat wear facets clearly distinguishable from the surrounding irregular texture produced by the polishing procedures. Slight parallel grooves in the antagonists sliding direction were evident on the second generation 3Y-TZP (Figure 2D) and on the 4Y-PSZ (Figure 2F) samples. For all the type III gold alloy specimens (Figure 2I,L), SEM observation suggested that the material was pushed towards both sides of the wear tracks rather than wearing and breaking away.

## 4. Discussion

In the present study, the in vitro wear resistance of different zirconia generations and a gold-based alloy opposing antagonistic cusps made out of the same restorative material was compared in order to simulate the clinical scenario of restored occlusal surfaces occluding in a full-mouth rehabilitation involving both upper and lower arches.

The null hypothesis tested had to be rejected, as significant differences in the wear behavior of the materials under investigation were revealed.

The gold-based alloy showed the highest wear values. As previously demonstrated [22,23], type III gold alloys display wear performances similar to those of human enamel, whose annual occlusal substance loss ranges from 15 µm (premolars) to 29 µm (molars) [25]. When performing prosthetic rehabilitations, clinicians should carefully consider the restorative material’s wear behavior in order to avoid jeopardizing the results of their therapies. On the one hand, in fact, a restorative material with an inadequate wear resistance could lead to occlusal contact loss (thus reducing the patient’s chewing ability and causing masticatory muscle fatigue) and altering of teeth–bone relationships and jaw relationships, eventually inducing functional and esthetic impairments [26,27,28,29,30]. On the other hand, a material with an excessive wear resistance could hinder the essential process of self-functionalization that physiologically every restoration might undergo, which helps protect teeth and prostheses from fractures and might prevent unpredictable occlusal interferences from ending up in gnathological problems. In these terms, due to its enamel-like wear behavior, type III gold-based alloys still might represent the gold standard for the replacement of masticatory surfaces. Nevertheless, on behalf of aesthetics, gold alloy use has progressively decreased over time, in favor of tooth-colored restorative materials [31,32] including monolithic zirconias. However, several in vitro reports have clearly shown the wear resistance of monolithic zirconia to be excessively different (significantly increased) compared to gold alloys or human enamel [21,33,34]; according to these authors, such a behavior should be considered less than ideal, especially with regard to the preservation of the patient’s occlusal balance.

Several zirconias with different chemical/crystallographic structure were evaluated in this study. Apart from 5Y-PSZ, which showed higher mean values for antagonist wear, wear depth, and volume loss, no significant differences were registered among the other zirconias. With a 900–1200 MPa flexural strength and a 6–8 MPa m^0.5^ fracture toughness [35,36], first generation 3Y-TZP is regarded as one of the most robust restorative ceramics [12]. Due to its alumina content and its crystallographic structure, however, first generation 3Y-TZP exhibits a considerable opacity, which makes matching natural teeth translucency very challenging [12,37]. These aesthetic limitations were gradually reduced by decreasing the amount of alumina from 0.25 to 0.05 wt% [18] (second generation 3Y-TZP) and increasing the yttrium oxide content (third generation 4Y-PSZ and 5Y-PSZ). A higher yttrium content (up to approximately 9.3 wt%/5 mol%) [38], in particular, results in a larger cubic phase, which limits residual porosities and light scattering at the grain boundaries and therefore allows a higher light transmittance [15,39]. At the same time, these chemical/crystallographic changes lowered the flexural strength and toughness of 4Y-PSZ and 5Y-PSZ [18,40] and deprived them of the renowned toughening mechanism by phase transformation typical of 3Y-TZP [15].

On the report of the findings of the present paper, the above-mentioned structural modifications of third generation zirconia (5Y-PSZ) seemed to significantly affect its wear resistance too. This seems in contrast with some previous studies [18,19,20], reporting that the reduction in flexural strength and toughness observed for third generation zirconias (especially 5Y-PSZ) is not necessarily followed by a concomitant decrease in wear resistance. This lack of consistency with previous papers could be imputable to relevant differences among the experimental study designs adopted. Rosentritt et al. [19] performed a pin-on-block wear test for 120,000 cycles. However, such a limited number of cycles might be insufficient to successfully detect any difference among wear-resistant materials such as zirconias and, for this reason, in the present study, the number of cycles was increased up to 360,000. Indeed, Zhang et al. [20] and Kwon et al. [18] increased the testing cycles up to 1,200,000 and 300,000, respectively, but they used a reduced load (20 N) and different antagonists, such as steatite spheres (Zhang et al.) and cone-shaped enamel cusps (Kwon et al.). Following a widely validated in vitro testing protocol (Ivoclar Method) [41], the study design of the present work instead provided a 50 N load and was the only one testing different zirconia generations and using the same material both for the specimen and the antagonistic cusp, thus obtaining useful results for clinicians dealing with restored occlusal surfaces occluding in a full-mouth rehabilitation. Nowadays, several resin composites and aesthetic ceramics exist that have proven to exhibit favorable enamel-like wear behavior [23]. Those materials provide all the clear advantages of an effective adhesive cementation [42], an adequate biocompatibility [43,44], and excellent aesthetic features due to their inherent translucency. However, their relatively limited flexural strength makes them not suitable for clinical situations requiring strong materials, in which zirconia might represent a more reliable choice. With the introduction of third generation zirconias, especially 5Y-PSZ, these mechanical advantages (although downsized) coexist with a remarkably enhanced translucency and aesthetics. The present study also showed a slightly more favorable wear behavior for 5Y-PSZ compared to the other monolithic zirconias. Nevertheless, its wear properties seemed to still be significantly different from a universally recognized gold-standard such as the gold-based alloy [4,21,22,23].

Advancements are therefore still needed in order to further improve 5Y-PSZ wear behavior, possibly without jeopardizing its current favorable aesthetic and mechanical features.

The present paper evaluated the wear features of modern high-translucent zirconia-based materials with all the advantages of an in vitro study following an experimental protocol that was well supported by the literature. The experimental design allowed the standardization of all the test parameters and the only qualitative factor under investigation was the type of dental restorative material. Care should be taken, however, when dealing with the translational process from in vitro results to clinical practice. Two-body wear tests, in fact, do not fully reproduce the complexity of the oral environment in terms of temperature, humidity, and pH features [45,46]. Moreover, both cylindrical specimens and cusp-shaped abraders used for two-body wear tests lack the elasticity provided by anatomical structures, such as periodontal ligament and alveolar bone, supporting the natural teeth under masticatory loads [45,47]. Thus, as a purpose for further research and with the aim of achieving a clinical validation of the present in vitro results, in vivo studies could be planned, designed to evaluate the intraoral wear of restorations made out of different zirconias, by comparing conventional or digital impressions [48] taken at the baseline and after months/years of clinical service [49,50,51].

## 5. Conclusions

Based on the findings of an in vitro model designed to test the two-body wear resistance of a restorative material opposing a cusp made out of the same material, the following conclusions were drawn:

1. The total vertical wear and total volumetric loss observed on first generation 3Y-TZP, second generation 3Y-TZP, and 4Y-PSZ were comparable;
2. All the tested zirconias showed statistically reduced wear values compared to the type III gold alloy;
3. The wear resistance of 5Y-PSZ was the closest to that of type III gold alloy, although it was still not statistically comparable.

## Figures and Tables

**Table 1:** Tested materials technical data provided by manufacturers.

Material	Lot. No.	Shade	Manufacturer	Technical Data
Zirconia Wieland Zenotec Zr Bridge	S27409	White	Wieland Dental + Technik GmbH & Co. KG, Munich, Germany	3 mol% yttria-stabilized tetragonal zirconia polycrystal
Katana Zirconia HTML	ECVXD	A1	Kuraray Noritake Dental Inc., Tokyo, Japan	3 mol% yttria-stabilized tetragonal zirconia polycrystal
Katana Zirconia STML	EDETQ	A3	Kuraray Noritake Dental Inc., Tokyo, Japan	4 mol% yttria partially stabilized zirconia
Katana Zirconia UTML	DQDUA	D4	Kuraray Noritake Dental Inc., Tokyo, Japan	5 mol% yttria partially stabilized zirconia
Aurocast8	15L0255	-	Nobil-Metal S.p.A., Villafranca d’Asti, Italy	Type 3 dental alloy with high gold content. Composition (W/W): Au = 85.4%, Ag = 9.0%, Cu = 5.0%, Pd = 1.0%, Ir = 1.0%

**Table 2:** Chewing simulator setting parameters.

Number of Cycles	360.000
Force	49 N
Height	3 mm
Lateral movement	−0.7 mm
Lowering speed	60 mm/s
Lifting speed	60 mm/s
Advanced speed	40 mm/s
Return speed	40 mm/s
Frequency	1.6 Hz

**Table 3:** Mean values (and standard deviations, SD) for antagonist wear, sample vertical wear, and sample volume loss. Same superscripted letters indicate no statistically significant differences.

Material	Antagonist Wear (mm)	Sample Vertical Wear (mm)	Sample Volume Loss (mm^3^)
UTML Zirconia Katana (Ultra-Translucent)	0.115 ^b^	0.073 ^a^	0.067 ^b^
	(0.021)	(0.023)	(0.029)
HTML Zirconia Katana (High-Translucent)	0.034 ^c^	0.012 ^b^	0.004 ^c^
	(0.027)	(0.007)	(0.003)
STML Zirconia Katana (Super-Translucent)	0.027 ^c^	0.018 ^b^	0.021 ^c^
	(0.016)	(0.004)	(0.013)
WB Zirconia (Conventional)	0.019 ^c^	0.015 ^b^	0.011 ^c^
	(0.008)	(0.006)	(0.007)
Aurocast 8	0.238 ^a^	0.082 ^a^	0.131 ^a^
	(0.078)	(0.017)	(0.055)

## Data Availability

The data presented in this study are available on request from the corresponding author.
